# Comparison of Physician Assistant and Medical Students' Clinical Reasoning Processes Using an Online Patient Simulation Tool to Support Clinical Reasoning (eCREST): Mixed Methods Study

**DOI:** 10.2196/68981

**Published:** 2025-12-01

**Authors:** Alistair Thorpe, Angelos P Kassianos, Ruth Plackett, Vinodh Krishnamurthy, Maria A Kambouri, Jessica Sheringham

**Affiliations:** 1 Institute of Epidemiology and Health Care, University College London, 1-19 Torrington Place, London, WC1E 6HB, United Kingdom, 44 02076798286; 2 Department of Population Health Sciences, Spencer Fox Eccles School of Medicine, University of Utah, Salt Lake City, UT, United States; 3 Cyprus University of Technology, Limassol, Cyprus; 4 Department of AI in Preventative Medicine School of Life Course & Population Sciences, King’s College London, London, United Kingdom; 5 Royal Free Hospital, Royal Free London NHS Foundation Trust, London, UK; 6 Institute of Education, University College London, London, United Kingdom

**Keywords:** clinical reasoning, medical education, primary care, educational technology, patient simulation tool, physician assistant, medical student

## Abstract

**Background:**

Clinical reasoning is increasingly recognized as an important skill in the diagnosis of common and serious conditions. eCREST (electronic Clinical Reasoning Educational Simulation Tool), a clinical reasoning learning resource, was developed to support medical students to learn clinical reasoning. However, primary care teams now encompass a wider range of professional groups, such as physician assistants (PAs), who also need to develop clinical reasoning during their training. Understanding PAs’ clinical reasoning processes is key to judging the transferability of learning resources initially targeted to medical students.

**Objective:**

This exploratory study aimed to measure the processes of clinical reasoning undertaken on eCREST by PA students and compare PAs’ reasoning processes with previous data collected on medical students.

**Methods:**

Between 2017 and 2021, PA students and medical students used eCREST to learn clinical reasoning skills in an experimental or learning context. Students undertook 2 simulated cases of patients presenting with lung symptoms. They could ask questions, order bedside tests, and select physical exams during the case to help them form, reflect on, and reconsider diagnostic ideas and management strategies while completing a case. Exploratory analysis was undertaken by comparing students’ data gathering, flexibility in diagnosis, and diagnostic ideas between medical and PA students.

**Results:**

In total, 159 medical students and 54 PA students completed the cases. PAs were older (mean 27, SD 7 y vs mean 24, SD 4 y; *P*<.001) and more likely to be female (43/54, 80% vs 84/159, 53%; *P*<.001). Medical and PA students were similar in the proportion of essential questions asked (Case 1: mean 70.1 vs mean 73.2; *P*=.33; Case 2: mean 74.6 vs mean 70.9; *P*=.27), physical examinations requested (Case 1: mean 54.7 vs mean 54.0; *P*=.59; Case 2: mean 69.3 vs mean 67.5; *P*=.59), bedside tests selected (Case 1: mean 74.4 vs mean 83.3; *P*=.05; Case 2: mean 47.9 vs mean 50.0; *P*=.69), and number of times they changed their diagnoses (Case 1: mean 2.8 vs mean 2.8; *P*=.99; Case 2: mean 2.8 vs mean 2.5; *P*=.81). Both student groups improved in their diagnostic accuracy during the cases.

**Conclusions:**

These results provide suggestive evidence that medical and PA students had similar clinical reasoning styles when using an online training tool to support their diagnostic decision-making.

## Introduction

Diagnostic error has been identified as the most common cause of avoidable harm in primary care [1]. Efforts to improve the clinical reasoning processes of health care staff who investigate and refer patients are critical for limiting the burden of disease by reducing diagnostic errors [2]. Clinical reasoning broadly encompasses the thought processes and strategies that underlie clinical judgments. Clinical reasoning is a core skill with relevance to many facets of clinical practice [3] and with a particular influence on clinicians’ judgments when attending to patients with symptoms that might be serious disease [4-6]. As a result, a need for formal education around clinical reasoning at all stages of the journey—from student to advanced professional—has been articulated [7,8].

eCREST (electronic Clinical Reasoning Educational Simulation Tool), an online patient simulation training tool, was developed for medical students to address this need [9]. It was tested with final-year medical students in UK medical schools, demonstrating good efficacy and receiving positive feedback from student testers about its value as an educational resource in improving clinical reasoning skills [10]. Interviews with medical student learners using eCREST, combined with analysis of their actions whilst using eCREST, demonstrated they displayed a range of data gathering strategies, with those more experienced on eCREST displaying more thorough data gathering strategies and identifying more essential diagnostic information [11].

Since eCREST was first developed, the landscape for primary care provision changed. In England, for example, primary care now encompasses a wider range of professionals beyond physicians and nurses to include pharmacists and social prescribers [12]. In addition, many primary care organizations now use physician assistants (PAs), with numbers working in primary care set to expand further [13]. PAs are a relatively new role in the United Kingdom, comparable to that of the PA in the United States, which have existed for over 50 years [14]. PA roles typically include taking patients’ medical histories, conducting physical exams, formulating differential diagnoses, and proposing management plans, including potential referrals [15]. Qualified PAs are master’s-level graduates who have completed a 2-year postgraduate course following a medical curriculum.

The expanded primary care team and the expansion of clinical roles have resulted in a need for clinical reasoning training suitable for a range of future clinical professionals, not just medical students. In a recent review of clinical reasoning across health professions, no resources were identified targeted toward PAs, as students or qualified professionals [7]. We adapted eCREST for PA students, in consultation with PA faculty, which involved ensuring the case description referred to PAs not just general practitioners (GPs). Adaptations were minimal but included changing the introduction at the start of cases to frame the cases for PAs and selecting from existing eCREST cases those that best fit the PA curriculum. The usefulness of eCREST as a meaningful and useful tool for PAs rests on the assumption that PAs approach clinical reasoning in a similar way to medical students. However, there is very limited understanding of how PA students in the United Kingdom acquire clinical reasoning skills, and whether they would exhibit similar reasoning processes to medical students. Insight into their reasoning processes while learning could be useful in designing multiprofessional education, for both current and future clinical professionals.

This exploratory study aimed to measure the processes of clinical reasoning undertaken on eCREST by PA students and compare PAs’ reasoning processes with previous data collected on medical students. It tests the a priori hypothesis that PA learners exhibit similar reasoning processes to those of medical students when using eCREST.

## Methods

### Theoretical Framework

In common with Young et al [16], we recognize that clinical reasoning is not a homogenous construct, nor is it understood in the same way by different individuals or professional groups. How clinical reasoning is defined has implications both for the focus of learning resources and how the effectiveness of such resources is evaluated.

Young et al [17] have mapped how different reasoning constructs and theories influence approaches to teaching and assessment, distinguishing between theories focused on acquisition of knowledge, knowledge organization, cognitive processes, or meta-cognitive processes. Informed by Plackett et al’s [18] review of the effectiveness of online simulated patient tools, eCREST was designed to focus on dimensions of clinical reasoning amenable to improvement and that are associated with common diagnosis-related biases in reasoning [9]. In this paper, therefore, we focus on clinical reasoning theories related to cognitive and meta-cognitive processes and display how these relate to eCREST in Table 1. Aligned with Young et al [16,17], we consider the process of clinical reasoning focusing on cognitive skills, with the outcome being reduction in cognitive biases supporting a goal of improving diagnostic reasoning.

**Table 1.** Application of clinical reasoning theory taken from Young et al [17] to the design and content of eCREST^a^.

Theoretical focus: good clinical reasoning requires...	Approaches to support students to develop clinical reasoning	Application to design of eCREST to develop reasoning skills	Reasoning measures derived from students’ completion of eCREST
...an ability to recognize relevant features in a clinical presentation and test hypotheses	Case-based learning using real or simulated cases	eCREST comprises a series of simulated patients through which students work.	Data gathering Diagnostic accuracy
...an ability to monitor one’s own processes for possible errors or biases and reflect on one’s own reasoning	Prompted reflection, clinical justification (eg, in patient notes)	eCREST seeks to influence learning through prompting reflection.	Diagnostic flexibility Reflections after the case
...sufficient knowledge base	Lectures and readings	eCREST was not designed to impart knowledge, but it starts with a knowledge quiz as a self-diagnostic, so students can revise key content before the case starts if needed.	Multiple-choice questions

a eCREST: electronic Clinical Reasoning Educational Simulation Tool.

### Design

We conducted a retrospective comparison of clinical reasoning processes and outcomes between medical and PA students using the eCREST online educational platform to teach clinical reasoning.

### Platform

eCREST is an online patient simulation training tool that has been tested with final-year medical students in medical schools in the United Kingdom [10,11]. eCREST [19] is proprietary software but the platform is available for use free to educational settings that are willing to contribute anonymized data and provide feedback to support its evaluation.

**Figure 1.**
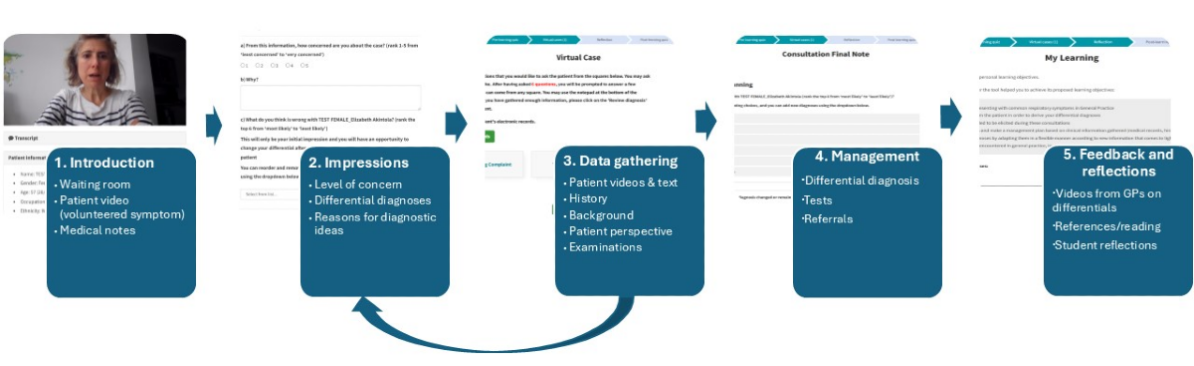
A student’s typical workflow through eCREST. Author JS is pictured in this figure. eCREST: electronic Clinical Reasoning Educational Simulation Tool; GP: general practitioner.

A workflow through eCREST is shown in Figure 1, with illustrative screengrabs from eCREST’s website during a simulated case. Briefly, students log on to the eCREST website and enter a virtual “waiting room.” They select a “patient” by clicking on a still of a patient actor and name, which opens a video of a patient actor who gives a brief account of the problem from the patient’s perspective for which they are seeking medical advice (Figure 1, panel 1). The student is then prompted to provide their ideas about likely diagnosis, through selecting 5 differentials from a long list of potential diagnoses; indicating on a scale of 1‐5 how worried they are about the patient, their ideas about likely diagnosis and typing as free text their reasoning behind their choices (Figure 1, panel 2). The student can then click on options to obtain further information—as videos or in text form—about the patient’s disclosed symptom, the presence or absence of other symptoms, and wider health (Figure 1, panel 3). At regular points during the “consultation,” the student is prompted to revisit their initial differential diagnosis choices. Finally, once students decide they have sought all the information they need, they are required to finalize their differential diagnosis and propose a management plan by selecting from further investigation and referral options and entering their rationale as free text (Figure 1, panel 4). Students can then access feedback, in the form of videos of GPs discussing why they prioritized certain differentials over other options. Once the case is completed, they are asked for their reflections and have the option to download a summary of their reasoning processes and GP feedback for each case (Figure 1, panel 5).

The content for the simulated cases included in this study was developed with clinicians, researchers, and patients informed by literature on diagnosis of common symptoms that could indicate lung cancer. A panel of GPs with a range of experience was convened to discuss and agree on the form of feedback. For each case, a GP in training developed the initial iteration of the case and proposed which information (ie, which questions, tests, and examinations) would be “essential” and what diagnoses would be most clinically important to consider. Each case was then reviewed by experts in primary care, diagnostics, and the disease systems as relevant to the case, to agree on the case content, including the options for differentials and the relevance of information sought.

### Recruitment

We worked with higher educational institutions (HEIs) in the United Kingdom to recruit participants. HEIs were sought with a diversity of teaching approaches and identified through personal contacts of the research team. Between 2017 and 2021, 4 universities providing PA programs and 3 medical schools used eCREST with their students. In the United Kingdom, undergraduate medicine courses typically have a duration of at least 5 years. In some cases, courses last 6 years, where students take an intercalated BSc between years 2 and 3. In the first years of training, students are typically mainly classroom-based. In later years of training, students have longer and more concentrated clinical placements, which include general practice, where they are required to be present in a general practice environment, primarily for educational purposes. During clinical placements in undergraduate medicine, students are mainly observing clinical interactions with opportunities for case-based discussion before and afterwards, though they may have some small direct care tasks under supervision, which increase in their volume and complexity, to prepare undergraduates for the foundation stage in their medical career, in clinical environments [20]. PAs typically undertake a 2-year master’s program, which covers similar content to medicine (eg, anatomy, physiology, and pharmacology) and provides exposure to clinical environments, including primary care, through clinical placements. Master’s graduates who pass the national Faculty of PA exams are then permitted to work as PAs throughout the United Kingdom’s National Health Service.

Medical students in this study in all 3 HEIs were in their final year of undergraduate training, PAs were in their final year of their master’s program. Students at each HEI were mainly recruited through advertisement in faculty newsletters, lecture “shout-outs,” social media, and tutor promotion between 2017 and 2018 and subsequently through the medical school promotion of eCREST via the teaching tutors. For medical students, participation was part of a feasibility trial. It was outside of timetabled lectures and entirely voluntary. However, for PA students, eCREST was introduced in relevant modules (eg, respiratory) by the study team in collaboration with teaching faculty during a scheduled session with the option for discussion of cases during later teaching sessions. Educators in each setting were consulted as to the best way to introduce eCREST to maximize possible educational value to students and educators and minimize burden to both.

As eCREST is an educational resource implemented within educational settings, the sample size was determined by uptake among students from the schools that had agreed to use eCREST. We did not perform a priori sample size calculations as part of this exploratory project. The content on eCREST was presented in English and accessed online by students.

### Procedure

On the advice of educators, UK medical students had access to 4 cases on eCREST, while PA students only had access to 2 cases. Analysis was undertaken on the 2 simulated cases that were common to all students. The cohorts completing each case were slightly different, but the results from both cases did not differ substantively, so we present data for the first case in the main text and the second case in Multimedia Appendix 1.

### Simulated Case

Students were presented with a 58-year-old female of Asian ethnicity presenting with chest pain (Case 1) and a 91-year-old male of White British ethnicity presenting with a cough (Case 2). Other information on the cases, including other symptoms experienced by the patient but not volunteered, was available to the student through selecting questions to ask the “patient” (to which they received a video reply) and by clicking on medical history or examination results. A full description of eCREST is available in Kassianos et al [9].

### Measures

When registering for eCREST, all students self-reported their program [medical student or physician assistant student], gender identity [male or female], and age [in y].

We selected quantitative outcome measures to align with three domains of clinical reasoning identified in the literature: data gathering, flexibility in thinking about diagnoses, and diagnostic accuracy [18]. We also included measures coded from student reflections obtained from free text data. These dimensions are:

1. Data gathering: students’ ability to elicit the necessary information to make an informed judgment.
2. Flexibility in thinking about diagnoses: students’ capacity to remain open-minded and to consider alternative views.
3. Diagnostic accuracy: students’ ability to identify the most relevant potential diagnoses for the patient.

Our previous work identified a gap in validated measures of clinical reasoning processes that are sensitive to change from short-term courses or training [10,11]. We have therefore developed our own measures of each of these dimensions, specific to eCREST.

In addition, we compare students’ free-text reflections about their own performance and transferable learning from the case to clinical settings.

### Data Gathering

Three measures were selected to reflect students’ case-specific clinical reasoning processes:

1. Essential questions asked: an expert panel of GPs determined that 20 of the 32 available questions were essential for coming to an informed diagnosis. For data analyses, this measure was calculated as (number of essential questions asked/20) × 100. For case 2, the expert panel identified 14 essential questions.
2. Essential bedside tests requested: an expert panel of GPs determined that for case 1, three of the 9 available questions were essential for coming to an informed diagnosis and for data analyses it was calculated as (number of essential bedside tests requested/3) × 100. For case 2, the expert panel identified 2 essential bedside tests.
3. Essential physical exams selected: an expert panel of GPs determined that for both cases, 6 of the 11 available physical exams were essential for coming to an informed diagnosis. For data analyses, this measure was calculated as (number of essential physical exams selected/6) × 100.

### Flexibility in Thinking About Diagnoses

Students were asked to select 6 specific diagnoses at the very beginning of the cases. They could then review their diagnoses at any point (with the option to change any of the diagnoses or leave it the same). They were also prompted to review their list of diagnoses every time they asked 6 questions, after they had asked 8 tests or exams, and then after they had asked for 2 more types of information (eg, question answer or a test or exam). We measured flexibility in thinking about diagnoses in 2 ways as the average number of times students changed diagnosis and the proportion of students who changed diagnoses at least once. We defined changing diagnosis as any one of the following: (1) introducing a new diagnosis they had not previously considered, (2) removing a diagnosis that they had previously considered, and (3) revising the order in which they prioritized the diagnoses they were considering.

### Relevant Diagnostic Accuracy

This was measured as the proportion of relevant diagnoses considered. An expert panel of GPs determined that, for both cases, 6 of the 13 available diagnoses were relevant to the patient case. This measure was calculated as (number of relevant diagnoses selected/6) × 100.

### Student Reflections

At the end of the case, students responded to four reflective questions on the case they had just completed, comprising: (1) What do you think you did well? (2) What do you think you need to improve? (3) What changed during the case? (4) What will you take forward from doing these simulated cases to your clinical work?

### Data Analysis

All analyses were conducted in R Studio (version 1.4.1106; Posit PBC) [21]. We compared whether the proportion of essential questions asked, bedside tests requested, physical exams selected, number of times changed diagnoses, and relevant diagnoses considered differed according to student type (medical student vs PA students). Qualitative responses to the four reflection questions were reviewed and coded to indicate whether the student’s reflection related to any of 6 themes, selected in part deductively based on eCREST’s goals of challenging cognitive biases, and in part inductively informed by ideas expressed in many of the student reflections: Knowledge, open-mindedness, awareness of cognitive biases, confidence or uncertainty, content of the case, no meaningful response.

These are retrospective analyses from a larger study of the eCREST tool. They were not preregistered, and a sample size calculation was not conducted. Significance was set at 2-sided *α*=.05 and we did not perform any adjustment for multiple comparisons in this exploratory study and instead report unadjusted effect sizes, 95% CIs, and raw *P* values [22].

### Ethical Considerations

This study was approved by University College London and the participating medical schools (15453/002). Invited student participants consented to take part and for their data to be analyzed as part of research on eCREST.

The undergraduate medical students in the United Kingdom were final-year students in a 6-year course, whereas the PAs were final-year students on a 2-year postgraduate training course, with an undergraduate degree in biomedical sciences. Information sheets and consent forms were shared through each student’s course lead and were also available for download on eCREST. Medical students participated as part of a trial and were offered compensation for completing cases (a maximum of £30 [US $40.54 at the time of the study; US $39.47 in 2025] in vouchers). PAs participated as part of their course. The information sheets confirmed that data would be stored in secure servers at University College London and used for research purposes and confirmed that their performance would not be shared with their course organizers.

## Results

### Participant Characteristics

A total of 654 students accessed and registered on eCREST during the study period. Of these, 213 students completed the patient case and were included in the analyses. Most were medical students (159/213, 75%), evenly distributed between 3 medical schools (School A: 51/159, 32.1%; School B: 60/159, 37.7%; School C: 48/159, 30.2%). Of the 54 physician assistant students, the majority came from one medical school (School D: 40/54, 74.1%; School E: 7/54, 13%; School F: 4/54, 7.4%; School G: 3/54, 5.6%; Table 2).

**Table 2.** Student institutions according to student type.

		Student participants	Completion^b^	
	Recruitment years^a^	Values, n (%)	Rate	Percent
Medical students				
School A	2017 and 2020	51 (32.1)	51/162	31.5
School B	2017‐18 and 2020‐21	60 (37.7)	60/168	35.7
School C	2017‐18 and 2020‐21	48 (30.2)	48/159	30.2
Physician assistant students				
School D	2021	40 (74.1)	40/126	31.7
School E	2020‐21	7 (13)	7/15	46.7
School F	2020	4 (7.4)	4/18	22.2
School G	2020	3 (5.6)	3/5	60

a All 2017 cohorts were part of a larger feasibility trial evaluation. Any other cohorts were recruited openly.

b Completion was calculated with all students who registered for eCREST from each school as the denominator.

The mean age of students in our sample was 25 (SD 5; range 19-48) years and most were female (n=127, 60%). The PA student group was older on average than the medical student group (*P*=.001) and contained a greater proportion of females (*P*=.001; Table 3).

**Table 3.** Student demographics overall and according to student type.

	Overall (n=213)	Medical students (n=159)	Physician assistant students (n=54)	Medical students versus physician assistant students	
				Values	*P* value
Age in years					
Mean (SD)	25 (5)	24 (4)	27 (7)	−3.48 (−5.53 to −1.42)^a^	<.001
Median (range)	24 (19-48)	23 (19-42)	25 (19-48)		
Age (years), n (%)					
19-24	142 (67)	117 (74)	25 (46)	—^b^	
25-34	56 (26)	35 (22)	21 (39)	—	
35-44	7 (3)	4 (3)	3 (6)	—	
45 and older	4 (2)	—	4 (7)	—	
Did not respond	4 (2)	3 (2)	1 (2)	—	
Gender, n (%)					
Female	127 (60)	84 (53)	43 (80)	10.9 (0.24)^c^	<.001
Male	86 (40)	75 (47)	11 (20)	—	

a Difference (95% CI).

b Not applicable.

c *X*2 value (φ). Degrees of freedom=1.

### Clinical Reasoning Measures

Full results are displayed in Table 4 and summarized below by outcome measure.

**Table 4.** Clinical reasoning outcome measures overall and according to student type.

	Overall (n=209)	Medical students (n=159)	Physician assistant students (n=54)	Medical students versus physician assistant students	
				Difference (95% CIs)	*P* value
Data gathering					
Essential questions				−3.1 (−9.4 to 3.2)	.33
Mean (SD)	70.9 (22.2)	70.1 (23.2)	73.2 (18.9)		
Median (range)	75 (0 to 100)	75 (0 to 100)	73 (10 to 100)		
Physical exams				−1.8 (−8.8 to 4.7)	.59
Mean (SD)	55.2 (19.2)	54.7 (18.6)	54.0 (21.1)		
Median (range)	50 (0 to 100)	50 (0 to 100)	50 (0 to 100)		
Bedside tests				−8.9 (−17.9 to 0.1)	.53
Mean (SD)	76.7 (29)	74.4 (29.1)	83.3 (28.8)		
Median (range)	100 (0 to 100)	67 (0 to 100)	100 (0 to 100)		
Flexibility in thinking about diagnoses					
Times changed diagnoses				0.0 (−0.5 to 0.5)	.99
Mean (SD)	2.8 (1.4)	2.8 (1.4)	2.8 (1.5)		
Median (range)	3 (0 to 7)	3 (0 to 7)	3 (0 to 6)		
0 times, n (%)	10 (4.7)	8 (5)	2 (3.7)	0.0 (0.03)^a^	.98
1 times, n (%)	33 (15.5)	23 (14.5)	10 (18.5)	—	—
2 times, n (%)	45 (21.1)	33 (20.8)	12 (22.2)	—	—
3 times, n (%)	60 (28.2)	49 (30.8)	11 (20.4)	—	—
4 times, n (%)	41 (19.2)	27 (17)	14 (25.9)	—	—
5 times, n (%)	20 (9.4)	17 (10.7)	3 (5.6)	—	—
6 times, n (%)	3 (1.4)	1 (0.6)	2 (3.7)	—	—
7 times, n (%)	1 (0.5)	1 (0.6)	2 (3.7)	—	—
Relevant diagnoses					
Initial				−8.2 (−12.6 to −3.7)	<.001
Mean (SD)	45.5 (14.8)	43.4 (14.4)	51.5 (14.2)		
Median (range)	50 (17 to 83)	50 (16.7 to 66.7)	50 (16.7 to 83.3)		
Change				5.3 (0.1 to 10.5)	.046
Mean (SD)	18.2 (16.7)	19.5 (16.6)	14.2 (16.64)		
Median (range)	17 (−17 to 67)	17 (−16.7 to 66.7)	17 (−16.7 to 50.0)		
Final				−2.85 (−7.5 to 1.8)	.23
Mean (SD)	63.6 (15.3)	62.9 (15.5)	65.7 (14.6)		
Median (range)	67 (17 to 83)	67 (16.7 to 83.3)	67 (33.3 to 83.3)		

a Represents *X*2 value with (φ) comparing students who changed diagnosis 0 times with those who changed at least 1 time by student type. Degrees of freedom=7.

#### Data Gathering

##### Essential Questions

Overall, students asked around 70% (14/20) of essential questions on average. We found no evidence of differences between PA students (mean 73.2, SD 18.9) and medical students (mean 70.1, SD 22.2) in the proportion of essential questions asked by each group (*P*=.33). Across all 20 essential questions, we found no evidence of differences between PA students and medical students in the proportion who asked each question (see Multimedia Appendix 1). For case 2, it was PAs (mean 74.6, SD 22.0) and medical students (mean 70.9, SD 22.9; *P*=.27; Multimedia Appendix 1).

##### Physical Exams

Overall, both medical and PA students selected around 55% (3.3/6) of essential physical exams on average. We found no evidence of differences between PA students (mean 54.0, SD 21.1) and medical students (mean 54.7, SD 18.6) in the proportion of essential physical exams selected by each group (*P*=.59). For case 2, it was PAs (mean 67.5, SD 17.9) and medical students (mean 69.3, SD 17.9; *P*=.59; Multimedia Appendix 1).

##### Bedside Tests

Overall, students selected around 77% (2.3/3) of essential bedside tests on average. On average, PA students (mean 83.3, SD 28.8) appeared to have selected a greater proportion of essential bedside tests than medical students (mean 74.4, SD 29.1), though the *P* value (*P*=.053) suggests there is only weak evidence for this difference. For case 2, it was PAs (mean 50, SD 50.0) and medical students (mean 47.9, SD 34.6; *P*=.69; Multimedia Appendix 1).

### Flexibility in Thinking About Diagnoses

#### Number of Times Students Changed Diagnoses

Overall, we found students changed diagnoses 2.8 times on average. We found no evidence of differences between physician assistant students and medical students in the number of times they changed diagnoses (*P*=.99). For case 2, the average number of times the diagnosis was changed was 2.5, and there was no difference between PAs (mean 2.8, SD 1.1) and medical students (mean 2.5, SD 1.1; *P*=.08).

#### Changed Diagnosis at Least Once

Overall, 203 of 213 (95%) students changed their diagnosis at least once. We found no evidence of differences in the proportion of PA students (151/159, 96%) and medical students (52/54, 95%) who changed their diagnosis at least once (*P*=.98). For case 2, all students changed diagnosis at least once.

### Diagnostic Accuracy

Overall, in their initial diagnosis, students included around 45% (2.7/6) of the relevant diagnoses on average. Compared with medical students (mean 43.4, SD 14.4), PA students (mean 51.5, SD 14.2) included a greater proportion of relevant diagnoses in their initial diagnosis (*P*<.001). For case 2, it was 43% (2.6/6) overall and did not differ between student groups (PAs: mean 41.5, SD 14.1 vs medical students: mean 44.4, SD 15.0; *P*=.19).

Both PA students and medical students had more relevant diagnoses in their final diagnosis than they did initially in case 1, with the overall proportion of relevant diagnoses included rising to 64% (3.8/6) on average overall. Notably, the average improvement was steeper among medical students (mean 19.5, SD 16.6) than PA students (mean 14.2, SD 16.7; *P*=.05). In their final diagnoses, both PA students (mean 65.7, SD 14.6) and medical students (mean 62.9, SD 15.5) were similar in the proportion of relevant diagnoses included (*P*=.23). For case 2 overall, it rose to 48% (SD 14.2; 2.9/6). Both PAs (mean 47.7, SD 13.9, +6.1 from initial) and medical students (mean 48.1, SD 14.3, +3.73 from initial; *P*=.84) were similar.

### Student Reflections

The reflections of both PA students and medical students were similar overall, with the following themes emerging from the data:

1. Knowledge-based: Reflections that focus on acquiring, applying, or deepening clinical or biomedical knowledge relevant to the case content.
2. Open-mindedness: Comments indicating a willingness to consider alternative diagnoses, questions, tests, exams, or approaches, and to revise initial assumptions.
3. Awareness of cognitive biases: Reflections that explicitly mention or imply recognition of cognitive biases (eg, anchoring, confirmation bias) and their potential impact on clinical reasoning.
4. Confidence: Statements related to the student’s self-assessed confidence in their clinical decision-making, diagnostic reasoning, or use of the eCREST tool.
5. Content: Reflections that comment on the structure, usability, or educational value of the eCREST tool itself, rather than the clinical case or reasoning process.
6. No meaningful response: Responses that lacked substantive reflection or were too brief or vague to be categorized under other themes.

Student responses are shown in Table 5.

**Table 5.** Reflection measures overall and according to student type.

	Overall (n=236), n (%)	Medical students (n=282), n (%)	Physician assistant students (n=46), n (%)	Medical students versus physician assistant students	
				Chi-square (*df*)	*P* value
What do you think you did well?					
Knowledge-based	8 (3)	8 (3)	0 (0)	0.61 (5)	.44
Open-mindedness	79 (28)	67 (28)	12 (26)	0.02 (5)	.89
Awareness of cognitive biases	2 (1)	2 (1)	0 (0)	0.00 (5)	.99
Confidence	7 (2)	7 (3)	0 (0)	0.44 (5)	.51
Case content	253 (90)	212 (90)	41 (89)	0.00 (5)	.99
No meaningful response	5 (2)	3 (1)	2 (4)	0.70 (5)	.40
What do you think you need to improve?					
Knowledge-based	49 (17)	46 (19)	3 (7)	3.65 (5)	.06
Open-mindedness	75 (27)	68 (29)	7 (15)	2.98 (5)	.08
Awareness of cognitive biases	21 (7)	21 (9)	0 (0)	3.23 (5)	.07
Confidence	13 (5)	12 (5)	1 (2)	0.23 (5)	.63
Case content	203 (72)	162 (69)	41 (89)	7.03 (5)	.008
No meaningful response	10 (4)	8 (3)	2 (4)	0.00 (5)	.99
What changed during the case?					
Knowledge-based	2 (1)	1 (0)	1 (2)	0.11 (5)	.74
Open-mindedness	91 (32)	73 (31)	18 (39)	0.84 (5)	.36
Awareness of cognitive biases	8 (3)	7 (3)	1 (2)	0.00 (5)	.99
Confidence	5 (2)	5 (2)	0 (0)	0.15 (5)	.70
Case content	154 (55)	132 (56)	22 (48)	0.72 (5)	.40
No meaningful response	55 (20)	44 (19)	11 (24)	0.39 (5)	.53
What will you take to your clinical work?					
Knowledge-based	17 (6)	14 (6)	3 (7)	0.00 (5)	.99
Open-mindedness	125 (44)	113 (48)	12 (26)	6.55 (5)	.01
Awareness of cognitive biases	17 (6)	17 (7)	0 (0)	2.37 (5)	.12
Confidence	12 (4)	9 (4)	3 (7)	0.19 (5)	.66
Case content	205 (73)	165 (70)	40 (87)	4.81 (5)	.03
No meaningful response	14 (5)	12 (5)	2 (4)	0.00 (5)	.99

Both groups of students focused most of their reflections on the content of the case (ie, describing their strategy or perceptions of how “accurate” they were) when asked what they did well, could improve, and would take into their clinical work. Recognizing the importance of being open-minded and flexible was also a common reflection by both groups of students.

However, there was moderate evidence of a difference in the learning students reported they would apply to their clinical work, in two respects. A greater proportion of medical student reflections related to open-mindedness and flexibility (48% vs 26%; *P*=.01). For example, one medical student reflected that, from now on, they would “always keep an open mind, not to be fixed on one diagnosis. Ask questions to rule out all possible differential diagnosis.”

A greater proportion of PA student reflections related to improving knowledge related to the clinical content of the case (87% vs 70%; *P*=.03). For instance, one PA student noted that they would take into their clinical work that they “have learnt which factors to consider” for the patient case, while another mentioned that “I will need to develop my knowledge of gastrointestinal differentials that cause respiratory-related symptoms.”

## Discussion

### Principal Results

This study yielded three key findings. First, we found no differences in data gathering through eCREST between medical students and PA students. Overall, both groups of students performed well on the proportion of essential questions asked (14/20, 70.9%), with scope for improvement on the essential physical exams selected (3.3/6, 55%). Second, we also found no differences between medical and PA students on our measures reflecting their flexibility in thinking about diagnoses. Third, we found that over the course of the cases, both PAs and medical students improved in the number of relevant diagnoses that they considered for the patients. These findings provided suggestive evidence that PAs and medical students used eCREST in similar ways to apply and develop diagnostic reasoning skills.

### Limitations

There are several methodological limitations to consider when interpreting the present findings. The use of just 2 simulated cases where differential diagnoses largely concerned respiratory and cardiovascular causes significantly limits the extent to which it is possible to generalize from these findings to cases seeking to develop diagnostic reasoning relevant to other bodily systems. The low sample size limited our ability to make strong statistical inferences or to adjust for possible confounders. Additionally, the medical students in our study self-selected to participate, and completion rates among those who registered were generally low. As we did not collect overall class cohort numbers, we were also unable to calculate response rates for all eligible students. Thus, there is a clear need for additional studies that can achieve greater recruitment and retention of student participants, particularly for the PA students, to yield larger and more representative samples. Notably, there was disproportionate representation of participants from School D, which may have introduced institutional bias and limited the generalizability of the findings across other PA programs.

The education context in which our two student groups also differed. As the medical students in our study had access to more cases (n=4) than their physician assistant counterparts (n=2), it is possible that they may have benefitted from being more familiar with the eCREST platform or from having practice with other cases. The context differed also in that medical students were completing cases before the COVID-19 lockdowns where online teaching was relatively unusual. In contrast, PAs were completing the cases in 2021, when online educational delivery was much more normalized. The difference in context, however, does not appear to have affected students’ responses to eCREST.

### Comparison With Prior Work

The findings of our study are broadly aligned with a review of performance of qualified PAs compared with physicians, which found performance on specific diagnostic tasks related to cancer was similar across both professional groups [23]. This review, however, is limited in its applicability to this study for two reasons. First, it was restricted to practicing clinicians, rather than those in training. Second, most of the literature included came from outside of the United Kingdom, in countries where primary care systems are not the same as the United Kingdom. As Barnhill et al found with respect to PAs in the United States, the role of PAs in primary care is different from the role of PAs in other health care settings [24].

We reflect also on the qualitative reflections of medical students and PAs in this study. We note this was a small exploratory study, so we are cautious about drawing firm conclusions regarding the underlying reasons for these differences but instead have reflected on key reflections from the groups. It is perhaps not surprising that a high proportion of PAs recognized in themselves a knowledge deficit (87% of PAs vs 70% of medical students; *P*=.03). At the point at which they commenced eCREST, they had experienced approximately 1.5 years of clinical training. In contrast, medical students would have had approximately 5 years of exposure to medical curricula. However, we interpret it as encouraging that a high proportion of both groups expressed a motivation to address knowledge gaps given the consistent evidence that knowledge deficits are a cause of diagnostic error [25].

It was interesting that nearly half of the medical students reported a need to develop open-mindedness and flexibility versus a quarter of PA students (48% vs 26%; *P*=.01). In light of evidence suggesting that confidence is a poor predictor of diagnostic accuracy [26], and that high levels of confidence lead to less information gathering, fewer changes in diagnosis, and biased evaluations [27], these reflections suggesting lower confidence and an increased awareness of the need to develop their reasoning could lead them to be better diagnosticians. The students’ reflections accord with the educational experiences of tutors using eCREST with a range of student groups who have observed that eCREST exposed students to clinical uncertainty and welcomed the stimulus provided by eCREST to discuss diagnostic complexity and uncertainty (unpublished data). We therefore consider it as encouraging for students’ future diagnostic reasoning that they identified the need for more open-mindedness as one of their key reflections.

### Conclusions

Developing students’ clinical reasoning skills is critical for improving patient care and reducing diagnostic error. These exploratory results provide suggestive and positive evidence that medical and PA students had similar clinical reasoning styles when using an online patient simulation training tool to support their diagnostic decision-making, and both groups reported some changes in their reasoning styles through using eCREST. As primary care teams widen to include a range of clinical professionals, further evidence is now needed to understand and compare how different clinical groups at all stages of training develop and apply essential clinical reasoning skills.

## Supplementary material

10.2196/68981 Multimedia Appendix 1 Data for the second case.

## Abbreviations

eCREST: electronic Clinical Reasoning Educational Simulation Tool
HEI: higher educational institution
PA: physician assistant
