# Supplementary material for: Comparison of Physician Assistant and Medical Students' Clinical Reasoning Processes Using an Online Patient Simulation Tool to Support Clinical Reasoning (eCREST): Mixed Methods Study
Source: JMIR Med Educ. 2025 Dec 1;11:e68981. doi: 10.2196/68981 (PMC12670056; doi:10.2196/68981)
Supplement: Multimedia Appendix 1 [file mededu-v11-e68981-s001.docx]

**Supplementary data file**

**Analyses of medical students and PAs on a second eCREST simulated patient case.**

**Supplemental table (1). Student demographics overall and according to student type. (simulated patient case 2)**

|  | | Overall, n=249 | Medical  students, n=192 | Physician associate  students, n=57 | Difference (95% CIs) | p value |
| --- | --- | --- | --- | --- | --- | --- |
| Age in yrs | | | | |  |  |
|  | Mean±SD, Median, [Range] | 24±5, 24, [19 to 53] | 24±4, 23, [19 to 53] | 27±7, 25, [19 to 48] | -3.40 (-5.34 to -1.46) | .001 |
| Age in yrs – no. (%) | | | | |  |  |
|  | 19 to 24 | 167 (67) | 142 (74) | 25 (44) | - | - |
|  | 25 to 34 | 66 (27) | 41 (21) | 25 (44) | - | - |
|  | 35 to 44 | 8 (3) | 5 (3) | 3 (5) | - | - |
|  | 45 and older | 5 (2) | 1 (1) | 4 (7) | - | - |
|  | Did not respond | 3 (1) | 3 (2) | 0 (-) | - | - |
| Gender – n (%) | |  |  |  | X^2^ value (φ) | p value |
|  | Female | 149 (59.8) | 104 (54.2) | 45 (78.9) | 10.2 (0.21) | <.001 |
|  | Male | 100 (40.2) | 88 (45.8) | 12 (21.1) | - | - |

| **Supplemental table (2). Clinical reasoning outcome measures overall and according to student type. (simulated patient case 2)** | | | | | | | |
| --- | --- | --- | --- | --- | --- | --- | --- |
|  |  | |  |  |  | Medical students  vs.  Physician associate students | |
|  |  | | Overall, n=249 | Medical  students, n=192 | Physician associate  students, n=57 | Difference (95%CIs) | p value |
| *Data Gathering* | | | | | |  |  |
|  | Essential Questions | | | | |  |  |
|  |  | Mean±SD, Median, [Range] | 71.7±22.7, 71, [0-100] | 70.9±22.9, 71, [0-100] | 74.6±22.0, 79, [14-100] | -3.7 (-10.3 to 2.96) | .273 |
|  | Irrelevant Questions | |  |  |  |  |  |
|  |  | Mean±SD, Median, [Range] | 35.3±30.1, 22, [0-89] | 34.8±30.4, 33, [0-89] | 36.7±31.5, 22, [0-88] | -1.8 (-11.2 to 7.55) | .702 |
|  | Physical exams | | | | |  |  |
|  |  | Mean±SD, Median, [Range] | 59.2±17.7, 50, [0-75] | 59.1±17.9, 50, [0-75] | 59.7±17.9, 50, [0-75] | -0.5 (-5.66 to 4.59) | .837 |
|  | Bedside tests | | | | |  |  |
|  |  | Mean±SD, Median, [Range] | 48.4±34.5, 50, [0-100] | 47.9±34.6, 50, [0-100] | 50.0±34.1, 50, [0-100] | -2.1 (-12.30 to 8.16) | .687 |
| *Flexibility in thinking about diagnoses* | | | | | |  |  |
|  | Times changed diagnoses | |  |  |  |  |  |
|  |  | Mean±SD, Median, [Range] | 2.5±1.1, 2, [1-7] | 2.5±1.1, 2, [1-6] | 2.8±1.2, 3, [1-7] | -0.3 (-0.69 to 0.03) | .075 |
|  |  | 0 times – no. (%) | - | - | - | - | - |
|  |  | 1 times – no. (%) | 47 (18.9) | 39 (20.3) | 8 (14.0) | - | - |
|  |  | 2 times – no. (%) | 82 (32.9) | 65 (33.9) | 17 (29.8) | - | - |
|  |  | 3 times – no. (%) | 72 (28.9) | 55 (28.6) | 17 (29.8) | - | - |
|  |  | 4 times – no. (%) | 38 (15.3) | 27 (14.1) | 11 (19.3) | - | - |
|  |  | 5 times – no. (%) | 8 (3.2) | 5 (2.6) | 3 (5.3) | - | - |
|  |  | 6 times – no. (%) | 1 (0.4) | 1 (0.5) | 0 (0.0) | - | - |
|  |  | 7 times – no. (%) | 1 (0.4) | 0 (0.0) | 1 (1.8) | - | - |
| *Diagnostic accuracy* | | | | | |  |  |
|  | Relevant diagnoses | | | | |  |  |
|  |  | Initial: Mean±SD, Median, [Range] | 43.7±14.8, 50, [17-83] | 44.4±15.0, 50, [17-83] | 41.5±14.1, 33, [17-67] | -2.84 (-1.45 to 7.13) | .193 |
|  |  | Change: Mean±SD, Median, [Range] | 4.3±12.1, 0, [-17-67] | 3.4±11.7, 0, [-17-67] | 6.1±13.6, 0, [-17-33] | -2.41 (-6.36 to 1.55) | .229 |
|  |  | Final: Mean±SD, Median, [Range] | 47.9±14.2, 50, [17-83] | 48.1±14.3, 50, [17-83] | 47.7±13.9, 50, [17-67] | 0.43 (-3.76 to 4.62) | .839 |
